# General practitioners’ perceptions about their role in current and future heart failure care: an exploratory qualitative study

**DOI:** 10.1186/s12913-019-4271-2

**Published:** 2019-06-28

**Authors:** Miek Smeets, Sofia Zervas, Hanne Leben, Mieke Vermandere, Stefan Janssens, Wilfried Mullens, Bert Aertgeerts, Bert Vaes

**Affiliations:** 1 Department of Public Health and Primary Care, KU Leuven, Kapucijnenvoer 33, blok j bus 7001, 3000 Leuven, Belgium; 2 Department of Cardiovascular Diseases, Universitair Ziekenhuis Gasthuisberg, KU Leuven, Leuven, Belgium; 3 Biomedical Research Institute, Faculty of Medicine and Life Sciences, U Hasselt, Hasselt, Belgium; 4 Department of Cardiology, Ziekenhuis Oost-Limburg, Genk, Belgium; 5 Institute of Health and Society, Université Catholique de Louvain, Brussels, Belgium

**Keywords:** Heart failure, General practice, Disease management, Qualitative research

## Abstract

**Background:**

A comprehensive disease management programme (DMP) with a central role for general practitioners (GPs) is needed to improve heart failure (HF) care. However, previous research has shown that GPs have mixed experiences with multidisciplinary HF care. Therefore, in this study, we explore the perceptions that GPs have regarding their role in current and future HF care, prior to the design of an HF disease management programme.

**Methods:**

This was a qualitative semi-structured interview study with Belgian GPs until data saturation was reached. The QUAGOL method was used for data analysis.

**Results:**

In general, GPs wanted to assume a central role in HF care. Current interdisciplinary collaboration with cardiologists was perceived as smooth, partly because of the ease of access. In contrast, due to less well-established communication and the variable knowledge of nurses regarding HF care, collaboration with home care nurses was perceived as suboptimal. With regard to the future organization of HF care, all GPs confirmed the need for a structured chronic care approach and envisioned this as a multidisciplinary care pathway: flexible, patient-centred, without additional administration and with appropriate delegation of some critical tasks, including education and monitoring. GPs considered all-round general practice nurses as the preferred partner to delegate tasks to in HF care and reported limited experience in collaborating with specialist HF nurses.

**Conclusion:**

GPs expressed the need for a protocol-driven care pathway in chronic HF care. However, in contrast to the existing care trajectories, this pathway should be flexible, without additional administrative burdens and with a central role for GPs.

**Electronic supplementary material:**

The online version of this article (10.1186/s12913-019-4271-2) contains supplementary material, which is available to authorized users.

## Background

In the last 20 years, clinical trials in the area of heart failure (HF) have provided a strong evidence base for medical and device treatments that have an effect on morbidity and mortality, especially for HF with reduced ejection fraction (HFrEF) [1–3]. This evidence is published in guidelines that are updated and disseminated to guide health care professionals in the deliverance of evidence-based HF care [2, 3]. However, providing guidelines alone is not enough to improve clinical practice. A paradigm shift is needed away from individual therapies to systems of care in which treatments are delivered [2, 3]. The goal of HF management is to provide a ‘seamless’ system of care, embracing both the community and the hospital [2]. This is translated in the development and implementation of comprehensive disease management programmes (DMPs). These DMPs have shown the potential to decrease HF readmissions by 30% and to decrease combined endpoints of readmission or death by up to 18% [2, 4–7].

Multidisciplinary care trajectories for diabetes and chronic kidney disease have existed in Belgium since 2014 [8], but structured, multidisciplinary approaches in the care of patients with HF are rare. Most HF DMPs have been hospital-initiated and target intramural HF care, together with the transition of care after an HF hospitalization [6, 7, 9]. From a community perspective, these HF DMPs target a minor part of the real-world HF population in primary care [10–12]. An integrated chronic care approach that includes population-based primary prevention, attention to multimorbid frail elderly persons with HF or palliative care is lacking in these DMPs [12–15]. A comprehensive DMP with a central role for general practitioners (GPs) could fulfil this need. However, previous qualitative research has shown that GPs have mixed experiences with multidisciplinary HF care [16].

The aim of this study was therefore to explore the perceptions that GPs have about their current role in HF care and their vision for the future organization of multidisciplinary HF care to be able to tailor interventions in a DMP to the needs of GPs as important stakeholders.

## Methods

An exploratory qualitative study design was chosen to outline the perceptions of GPs about their role in the current and future care of their patients with HF. Qualitative research is the best way to acquire insight into the experience and opinions of participants. Face-to-face semi-structured interviews were performed because a number of relevant research topics had used this format in previous research [17]. The study was submitted to and received approval from the ethical committee of the University Hospital Leuven (mp05169). All GPs provided written informed consent prior to participation. The consolidated criteria for reporting qualitative research (COREQ) checklist was used as guidance to report our research findings [18].

### Participants and recruitment

GPs with various backgrounds (Table 1) from the Limburg region (Flanders, Belgium) were interviewed, aiming for maximal variation in characteristics by means of targeted selection (*purposive sampling*) [17]. Variation was sought regarding sex, years of experience, practice type, experience in working with different disciplines and distance to the hospital (Table 1). Years of experience was reported as a range (0–5, 5–10, 10–15, 15–20, 20–25, 25–30, 30–35, 35–40, ≥40). GPs were eligible to participate if they were practising GPs, working in the Limburg region and Dutch speaking. GPs were invited for an interview by phone; we did not remunerate participation in the study and asked for a reason in case of denial to participate. We aimed for 10 to 15 interviews because this was a reasonable number to reach data saturation.

**Table 1:** Characteristics of the participating GPs

GP (number)	Years experience (range)	Practice type (number of GPs)	Other disciplines in practice	Collaboration with hospital and distance (travel distance by car in minutes)
1	35–40	Solo	/	ZOL Genk (9)
2	0–5	Group (6 + trainee)	nurse, dietician, psychologist	ZOL Genk (14)
3	20–25	Group (3 + 2 trainees)	/	MZNL Overpelt (10)
4	25–30	Group (3)	physiotherapist	Sint-Franciscus Heusden-Zolder (13) ZOL Genk (16) Jessa Hasselt (19)
5	15–20	Group (2 + 1 trainee)	/	ZMK Maaseik (9) ZMK Bree (18) ZOL Genk (18)
6	0–5	Duo	/	Sint-Franciscus Heusden-Zolder (14) MZNL Overpelt (25) Jessa Hasselt (26)
7	0–5	Group (3)	/	Sint-Franciscus Heusden-Zolder (7) ZOL Genk (17)
8	25–30	Duo	/	Sint-Trudo ziekenhuis (5) Jessa Hasselt (22)
9	30–35	Group (6 + trainee)	/	ZOL Genk (11)
10	≥40	Solo	/	AZ Vesalius Tongeren (9)
11	25–30	Group (3 + trainee)	/	ZMK Bree (7) MZNL Overpelt (20)
12	0–5	Group (6 + trainee)	nurse	Jessa Hasselt (6)
13	10–15	Group (5 + trainee)	dietician, psychologist	Jessa Hasselt (5)

### Data collection

Two of the authors (HL and SZ) conducted the face-to-face semi-structured interviews between March 2015 and February 2016. One of the authors functioned as interviewer, the other as observer making field notes. This approach was chosen to maximize the familiarity of both researchers with each interview and each context. Both authors are female and were GP trainees at the time in the Limburg region. They were familiar with some of the interviewees within a professional context. MS and MV trained both authors in conducting interviews, and after each interview the interview format was evaluated and adapted to aim for maximal quality. The interviews took place in the participating GP’s practice. All participating GPs gave informed consent prior to the interview. All interviews were audio-recorded. A topic list was used that was based on the literature to structure the interviews, and we appraised and adapted the list where necessary [Additional file 1: Topic list]. The topic list and the interviews were in Dutch, as this was the language most familiar to the interviewers and the interviewees. The results were translated into English by a professional language editor. Interviews were conducted until data saturation was reached. Data saturation was defined as the moment when the previous two interviews no longer contributed any new elements and when a certain category had been exhaustively described in all its dimensions and variations. This means that conducting additional interviews would no longer provide new insights.

### Data analysis

Each interview was literally transcribed, including both verbal and non-verbal signs (*conversation analysis*) [17]. The interviews were conducted in cycles of three to four and were then processed. After eleven interviews, two more interviews were planned to reach data saturation.

The principles of the Qualitative Analysis Guide of Leuven (QUAGOL) were followed in the data analysis [19]. The procedure consisted of two parts: (i) a preparatory part using pen and paper and (ii) the actual coding process using NVIVO 10 software (QSR International, Melbourne, Australia). Each part, in turn, consisted of five phases that were processed dynamically.

During the preparatory part, the aim was to become as familiar as possible with the interview data in order to compile a list of concepts as a starting point for the actual coding process in NVIVO 10. First, two authors (HL and SZ) formulated the context and essence of each interview separately and then discussed this together. A conceptual scheme was then drafted for each interview. The usability of this scheme was monitored by repeated comparison with the interview. Additionally, conceptual schemes of the various interviews were analysed and compared to eventually compile a list of concepts.

Two authors (HL and SZ) performed the actual coding process using NVIVO 10 software. In the first step, the data was coded by linking each fragment of text to one of the concepts from the list. The concepts thus served as an initial coding tree in the programme. The usability of the codes and concepts were evaluated by the team (HL, SZ, MS, BV and MV) and adapted where necessary. In the last step, two researchers (HL and SZ) separately distilled the storyline from the findings and concepts. Next, this was discussed between them and then submitted to the research team (HL, SZ, MS, BV) to reach a consensus.

The descriptive themes were created by merging codes of the same nature (inductive method). Analytical themes were derived from these descriptive themes via a thorough group discussion. Investigator triangulation was used to obtain more reliable results in almost all stages of the analytical process by means of intense collaboration of different members of the research team, all with their own backgrounds.

## Results

### Participants and recruitment

A total of 13 out of the 17 GPs invited to participate agreed to be interviewed (Table 1). Lack of time was given as the reason by all those who declined to participate. Unfortunately, all four practices that declined participation were solo practices, leading to a relative underrepresentation of this practice type (2/13). However, a large variation within (group) practices was still obtained, and the GPs varied by age, years of experience and location. The interviews lasted between 37 and 60 min.

### Theme 1: GPs’ perceptions about their current role in HF care

A comprehensive overview of all identified themes is provided in Table 2. In this table, the themes are organized into HF-specific factors, patient factors (non-modifiable) and physician and contextual factors (modifiable) to facilitate the recognition of modifiable factors.

**Table 2:** Thematic matrix

Theme 1: GPs’ perceptions about their current role in HF care				
	HF-specific factors	Patient factors	Physician factors	Contextual factors
Prohibiting factors				
*General HF care*	• HF is perceived as labour-intensive	• Difficulties associated with comorbidities, age and polypharmacy	• Lack of experience • Lack of confidence	• Lack of time • Administrative burden
*Diagnosis*	• Non-discriminating symptoms and signs in chronic HF	• Reluctance in diagnosis of older patients	• Lack of awareness for HF	• Lack of availability of diagnostic tests in primary care
*Treatment*		• Difficulties associated with comorbidities and polypharmacy • Limited compliance with taking diuretics • Reluctance to treat older patients	• Lack of up-to-date knowledge • Lack of experience	
*Education*		• Lack of patient compliance with dietary restrictions	• Lack of motivational interviewing skills	• Lack of time/HF education is experienced as time-consuming
Theme 2: Roles of GP within a multidisciplinary team				
*Collaboration with cardiologists*				
Prohibiting factors			• Resistance to work with cardiologists that do not share patients or who do not communicate • Specialist care leads to fragmented care	• Transition of care after discharge from hospital should be improved • Lack of two-way communication about patients
Facilitating factors			• Preference for cardiologists that share care, are easily accessible, and give advice for follow-up in discharge letters • Informal contact with specialists outside of practice eases collaboration	• Easy access to cardiologists - Telephone advice to GPs - Short waiting lists for patients • Fast and qualitative feedback by letter after cardiologist consultation
*Collaboration with nurses*				
Prohibiting factors			• Lack of trust in the nurse competencies • Nurses lack knowledge about HF alarm symptoms	• Difficult communication about patients • Lack of role clarity in telemonitoring projects
Facilitators			• Positive previous experiences	
*Future organization of multidisciplinary HF care*				
Care pathway for HF		• Needs to be flexible/patient-centred	• Meets the need for support in staying up-to-date/doing the right thing • Fear of being overlooked • Fear of fragmentation of care	• Central role for GP • No additional administration • Structured guidance, not obligatory
Collaboration with specialized HF nurses			• Fear of being overlooked • Fear of fragmentation of care • Lack of knowledge about HF nurses	• Preference for all-round general practice nurse • Need for financial support • Need for support in education of general practice nurses

### GPs’ general ideology

The GPs’ perceptions about their current and future role in HF care could be understood by studying what matters to them in their profession.

GPs saw themselves as the central figure who monitors the overall therapy and the patient’s quality of life. They felt best positioned for this role because of their overall view of the patient in his or her context. Therefore, they expressed the wish to take up a central role in the care for patients with HF.

*“A central role, even if the patient sees consultant A and then sees a nephrologist a few days later. Imagine consultant A prescribing something that would not be good for the kidneys … Wouldn’t we at least point this out? So, a central role. It’s our job to manage the patient’s medical records, the overall medical record”. (GP 4).*

This central role led to a close relationship between doctor and patient and was felt to be a great source of professional joy. However, due to increasing work pressure, some doctors expressed the fear that this central role would no longer be tenable in the future.

Additionally, GPs considered it very important to provide tailored care to patients with HF. Advice and management were adapted to the patient’s condition and wishes whenever possible (stratified care). Younger, fitter patients were standardly referred to a cardiologist, whereas geriatric, vulnerable and cognitively less able patients were more often kept in their own care (Table 2).

*“And in a long-term care facility, it does mean that all of these patients … yes … how shall I put it... That your first concern is controlling symptoms and making sure that they remain comfortable. And not pulling out all the stops for these patients anymore. So the follow-up is somewhat more limited than that of young, active patients with heart failure.” (GP 7).*

### GPs’ specific job description in HF care

GPs consider diagnosis, referral and follow-up as their specific tasks in HF care. With regard to diagnosis, ‘*awareness’* was frequently mentioned, in other words, recognition of HF. A cardiologist needs to confirm the HF diagnosis. However, GPs reported that it is their job to turn the cardiologist’s patient management plan into practice.

*“I think that as a GP I generally have a clear picture of who and what the patient is, what they know and can do. I have a good view on what matters socially for the patient. I think my job is to actually transfer the theory into practice, into concrete practice.” (GP 13).*

GPs reported that follow-up of patients with HF included monitoring the clinical situation, maintaining an overview of medication (interactions, comorbidity, adaptations in case of decompensation) and educating the patient. It was frequently reported that the latter is time-consuming and not always carried out by the doctor (Table 2). Their experience and personal interests determined the degree to which GPs truly engage in patient education.

*“There usually isn’t enough time to concentrate a lot on the non-medication part of the treatment. That’s at least how I experience it. My colleague is rather more dynamic in these matters so he easily extends his consultations to half an hour or three quarters to get things done. But I don’t, I stop because of the heavy workload.” (GP 7).*

### Prohibiting and facilitating factors experienced by GPs in HF care

Overall, GPs indicated that they were satisfied with their current role in the care of patients with HF. However, some factors were perceived as prohibiting (Table 2).

#### HF-specific factors

GPs struggled with the diagnosis of HF since symptoms and signs were perceived as non-discriminating for HF. One GP commented on the use of natriuretic peptides to overcome this diagnostic uncertainty but perceived them as “guiding but not conclusive” and expressed the need for a more conclusive diagnostic approach in primary care.

#### Patient factors

Additionally, GPs encountered difficulties associated with comorbidities and polypharmacy, especially in elderly patients. Furthermore, several GPs saw older age as a consideration for a less aggressive approach. Moreover, GPs reported difficulties to motivate patients and informal caregivers to follow therapy and lifestyle advice, often due to a lack of insight.

*“Some patients you can’t get in a stable state. For example, patients that do not take their diuretics when they go to a shop or have to do something else. You cannot convince them that it is important to take them daily. They believe ‘it is medication to pee’ and they don’t relate it to their heart problems. It is difficult to explain such things because they don’t feel like changing.” (GP 12).*

#### Physician factors

HF management was perceived as difficult, labour intensive and something you had to learn by doing. Younger doctors reported a lack of experience and confidence as a prohibiting factor, while older doctors reported confidence through experience and a more active personal role in adapting HF care. Younger doctors tried to compensate for their lack of experience by consulting more experienced GP colleagues or cardiologists by phone. Several GPs, irrespective of age, found it difficult to stay up-to-date with HF treatment options because there have been many changes in recent years.

*“The fast development of new medications that I don’t know whether they are better or not or if a medication is outdated. What do I do?” (GP 2).*

#### Contextual factors

GPs experienced a heavy workload, and consequently a lack of time, as one of the most important limiting factors. Additionally, they felt the increased administrative burden stood in the way of their clinical work.

### Theme 2: GPs’ role within a multidisciplinary team

GPs reported that cardiologists and home care nurses are the most important partners in the care for patients with HF.

### Collaboration with cardiologists

#### Cardiologist role in HF management

GPs saw cardiologists as the professionals who diagnose HF, provide a technical outline of the situation, and (if not yet done so by the GP) draw up a management plan and start the patient on medication. Patients then regularly consulted the cardiologists for follow-up. Unstable patients were referred for an extra consultation or, in case of emergencies or clinical deterioration, GPs consulted cardiologists by phone. Routine communication was by letter.

*“If I notice I’m losing this man here for one or other reason, he’s decompensating further, then I quickly pick up the phone and ask, ‘what shall I do with him?’” (GP 4).*

#### Prohibiting and facilitating factors in collaboration

Collaboration with cardiologists was described as smooth. Overall, GPs were satisfied with the communication because cardiologists were easily approachable. The availability of cardiologists by phone was considered positive, and waiting times for a cardiology consultation were considered reasonable. GPs tended to choose preferred partners to work with based on the quality of communication, ease of access, personal connections and especially perceptions of shared care. Cardiologists known to take over care for patients instead of sharing care were avoided. By working with preferred partners, some obstacles for interdisciplinary collaboration were overcome.

*“Some cardiologists are GP-friendly, others are more of the kind that if you refer patients to them you don’t see them anymore. But after a while you’ve figured this out and then you refer your patients to those that alternate between consultations with them and us.” (GP 9).*

However, transition of care after discharge from the hospital could be improved.

*“At the time of discharge, they should have contacted me by phone, because this is an elderly lady and heart failure plays an important role here, but social problems are also quite important. This lady is now at home, and socially this is not good, which in turn hinders the treatment of heart failure.” (GP 13).*

Additionally, it was reported that cardiologists do not always make full use of GPs’ knowledge of patients.

*“Secondly, I think that before they started her on diuretics they could have asked the treating physician about it; ‘Have you experienced this before? Have there been any problems … ’” (GP 13).*

Furthermore, GPs noticed that specialist care in general often led to fragmented care instead of integrated care. Cardiologists who evaluated the patient’s comorbidities beyond their own discipline were appreciated the most.

### Collaboration with nurses

#### The role of primary care nurses in HF management

In Belgium, specialist HF nurses only work in hospitals. GPs generally collaborated with primary care nurses who visit patients at home (home care nurses) or who work in long-term care facilities. The nurses were primarily perceived to have a warning function. They are in close contact with patients and they alert the GP in case of changes in the patient’s overall condition.

#### Prohibiting and facilitating factors

Collaboration with home care nurses was not always positively perceived. Communication problems and the insufficient medical HF knowledge of nurses were most often cited as prohibiting factors. Communication booklets were reported to be chaotic and unclear, and electronic platforms were still not easily available. An extra concern of some GPs was the recent development of telemonitoring, with nurses wiring patient parameters to GP practices on a daily basis. They stressed the importance of maintaining the nurses’ warning function, because they felt GPs could not be expected to readily process this multitude of electronic information. Insufficient HF knowledge of the home care nurses was apparent from the inadequate assessment of the degree of medical urgency. This led to some nurses foregoing their critically important signalling role to the GP.

*“I think home care nurses sometimes get the instructions to immediately call for the doctor whenever they see a swollen leg, so that makes them a bit too concerned I think, there it is.” (GP 13).*

*“Some nurses really need to be told repeatedly: please, these are our alarm symptoms, alert us.” (GP 5).*

As with the preferred partnerships with cardiologists, GPs also selectively referred patients to some home care nurses due to historically better collaborations with them. A stimulating factor to do so was trust in the caregiver’s capacities.

*“You more or less choose your nurse, don’t you. Certainly those self-employed nurses that you can rely on.” (GP 4).*

### Future organization of multidisciplinary HF care in Belgium

When GPs reflected about the future organization of multidisciplinary HF care in Belgium, a few concepts were discussed such as the implementation of a care pathway or care trajectory for HF and the collaboration with specialized HF nurses in primary care.

### A care pathway or care trajectory for HF

The majority of GPs expressed the need for more protocol-driven HF care. Receiving instructions or recommendations from hospital-based caretakers from regional health authorities on managing HF patients was perceived as a welcome support.

*“In heart failure, you could say … if there was a protocol … for example hospital-driven, if the patient is diagnosed, you should do this and a primary care nurse should do that … Then you could easily delegate tasks …” (GP 2).*

However, a few prohibiting factors were expressed.

First, GPs strongly preferred that care should remain patient-oriented. They considered other existing care trajectories to be too rigid and overburdened with administration. Therefore, they stressed the hope that, contrary to care trajectories, care pathways should be flexible and should not result in more administrative work.

*“But I think they should make it possible for care to be better adjusted to the patient … so that there is a standard trajectory but that you can say, OK, we can make a ‘light’ version of it, or an ‘extended’ ‘luxury’ version of it, and also check a little bit what the patient really needs.” (GP 6).*

Second, GPs expressed the fear of being overlooked, and third, the concern that another care pathway for multimorbid patients with HF would lead to fragmented care instead of integrated care. To overcome these obstacles, it was considered crucial to solidify the central role of GPs in care pathways, allowing HF management to be tailored to the patient’s needs and ensuring continued monitoring by GPs of their patients’ overall therapy.

### Collaboration with specialized HF nurses in primary care

To cope with the increasing workload, the feeling predominated that some tasks in HF care could and should be delegated, such as patient education and regular follow-up of parameters. However, regarding the role of specialized HF nurses in primary care, the same concerns as above were expressed, including the fear of being overlooked and of care fragmentation. Many GPs also admitted that they had little knowledge about the tasks a specialized HF nurse could fulfil. Therefore, delegating tasks to an all-round general practice nurse, capable of managing all chronic diseases, was preferred.

*“If cardiologists do everything themselves and nurses do everything themselves, then we lose our role, don’t we? So that’s perhaps something that we GPs are a bit worried about as to what it will happen in the future.” (GP 9).*

*“Well, I would be positive about a nurse in the practice. But then again, it comes down to the same thing. We’re not cardiologists, are we? We’re GPs. So a nurse specifically for heart failure, I think that what this nurse monitors, somebody else can monitor too, and then I’ll interpret the data during the consultation. How many nurses would you need then in your GP practice?” (GP 8).*

To evolve to such a care model, GPs expressed the need for more support both financially and with regard to the education of nurse practitioners.

## Discussion

### Summary of results

The GPs expressed the fear that their central role is coming under pressure because of the increasing workload they experience. Additionally, HF specifically is regarded as labour-intensive and difficult to manage due to patient comorbidities and polypharmacy. Furthermore, some (mostly younger) GPs experience a lack of knowledge and confidence in the management of HF. GPs report that the current multidisciplinary collaboration is limited to cardiologists and primary care nurses and is not structured. The need for a multidisciplinary chronic care approach for HF was confirmed and would preferentially be channelled in a structured care pathway that should be flexible and patient-centred, with a central role for GPs and without additional administration. An all-round general practice nurse was viewed as the preferred partner to delegate tasks to in the management of HF patients.

### Comparison with the literature

Hancock et al. stated in 2014 that barriers to the accurate diagnosis and management of HF did not change in the past 10 years [20]. The same prohibiting factors indeed recur in our study [16]. GPs still experience HF as a challenging diagnosis because of difficulties in managing multimorbid, elderly patients, insufficient up-to-date medical knowledge about HF, a lack of time and a high administrative burden [16, 20]. Hancock et al. wondered why so few physicians considered the use of guideline-recommended natriuretic peptides as a solution for their diagnostic uncertainty in HF [2, 16, 20]. This implementation gap seems to remain [16, 20]. In our study, only one GP commented on the use of natriuretic peptides in the diagnosis of HF but experienced the use of these peptides as insufficiently conclusive. However, GPs in Belgium have little experience with natriuretic peptide testing, since it is not reimbursed.

Regarding interdisciplinary collaborations, our findings are in line with international research [16]. However, due to local health care organization, important differences are also obvious. First, access to fast and nearby echocardiography is almost ubiquitous in Belgium, facilitating collaboration between cardiologists and GPs [16, 21–25]. However, GPs valued even more the fact that they could easily contact cardiologists by phone for advice about patients. The transition of care after hospital discharge was particularly mentioned. This was an important remark because the impact of a follow-up visit within 30 days after discharge on death and readmission has been proven [4, 26, 27]. Second, in our study, GPs experienced the collaboration with home care nurses as troublesome due to communication difficulties and the perception that nurses lacked the knowledge required to adequately fulfil their warning function. Third, the GPs had little knowledge about specialist HF nurses. This is in contrast with international studies that reported more positive experiences with regard to GP-nurse collaborative practice [24, 28, 29].

### Implications for the design of an HF disease management programme

First, an interesting suggestion by the GPs themselves was to stimulate the evolution towards more protocol-driven care for HF. They expressed their interest in a care pathway for HF. This pathway should contain clear instructions for patient follow-up to standardize the care of HF patients; however, the pathway should have enough flexibility to keep it patient-centred. Patient-centred care was a key value for most of the interviewed GPs, as it should be [30]. However, patient-centeredness could also lead to too much variability in care and to the ‘benign neglect’ of care for the elderly [31]. Therefore, the balance between protocol-driven guideline-based care and patient-centred care could be improved by a (national) care pathway for HF.

Second, GPs reported that their increasing workload will force them to organize their practice differently in the future and to delegate more tasks in order to maintain the same quality of care. This was acknowledged by the Belgian Healthcare Knowledge Centre in 2012 [15]. However, the question remains—who is the preferred partner to delegate tasks to in primary care HF management and how should this be organized? The role of specialist HF nurses in the hospital is evident and well-described; however, their role in primary care is more controversial [2, 32, 33]. The interviewed GPs saw an all-round general practice nurse as their preferred partner as opposed to specialist HF nurses because of a fear of being left out and a fear of care fragmentation. The latter may be a justified fear. For the majority of HF patients, the optimization of disease management by primary care personnel (GPs and primary care nurses) will be sufficient [14, 15, 34]. A crucial factor herein is education and support of the primary care personnel, a role suitable for specialized HF nurses. Additionally, for a small number of highly complex HF patients (5–10%), case management by a specialist HF nurse will be necessary. Specialized nurse-led home-based care has only been proven beneficial for patients at highest risk for (re) hospitalization [5, 33–37]. Hence, a model in which an appropriated number of specialized HF nurses educate and assist a large number of GPs, primary care nurses and patients could work, as shown by a successful and cost-effective UK initiative [37].

### Strengths and weaknesses

A strength of the current study is the use of the QUAGOL method as guidance for the data analysis, leading to a thorough reading and rereading of the data and discussions between the researchers on the data needed to capture the essence of each interview before starting the actual coding process. Additionally, only four out of 17 GPs refused to participate, leading to a high response rate, possibly because both interviewers are GPs themselves. The latter may also have put the GPs at ease and stimulated them to open up. However, all four GPs that refused to participate were solo practices and indicated a lack of time, leading to a relative underrepresentation of solo practices (2/13). Another limitation could be the limited experience of the interviewers with qualitative research. However, this was compensated for by a thorough training in semi-structured interview techniques and qualitative data analysis. Additionally, investigator triangulation was used to limit this bias as much as possible.

## Conclusions

The GPs in this study value their central role in HF care but notice that future practices should adapt to the increasing workload. Collaboration with cardiologists is perceived as smooth, while collaboration with home care nurses is often perceived as suboptimal. GPs strongly advocate a structured care pathway for HF that is flexible and acknowledges a central role for the GP without additional administration. They support the delegation of specific tasks in HF management to other health care professionals to improve communication of early warning signs and enhance patient education. A model in which an appropriate number of specialized HF nurses educate and assist a large number of GPs, primary care nurses and patients is favoured and should be further studied.

## Additional file

Additional file 1: Interview topic list. (DOCX 13 kb)

## Data Availability

The datasets used and analysed in the current study are available from the corresponding author on reasonable request.

## Abbreviations

COREQ: Consolidated criteria for reporting qualitative research
GPs: General practitioners
HF: Heart failure
QUAGOL: The Qualitative Analysis Guide of Leuven
UK: United Kingdom
