# Supplementary material for: General practitioners’ perceptions about their role in current and future heart failure care: an exploratory qualitative study
Source: BMC Health Serv Res. 2019 Jun 28;19:432. doi: 10.1186/s12913-019-4271-2 (PMC6599228; doi:10.1186/s12913-019-4271-2)
Supplement: Supplementary file 1 — Interview topic list. (DOCX 13 kb) [file 12913_2019_4271_MOESM1_ESM.docx]

**Additional file 1: Topic list**

Translated version (from Dutch to English):

Opening question: Can you recall your latest heart failure diagnosis? How did you experience the care for this patient?

1. Which role do GPs wish to take in the care for heart failure patients?
   1. In general
   2. More specifically:
      1. In pharmacological treatment
         1. Starting medication?
         2. Follow-up?
         3. Titrating medication dosages?
      2. In non-pharmacological treatment
         1. Patient education?

Which barriers do they experience in taking up this role in HF care?

What could facilitate taking up the desired role in HF care?

1. What is the role of GPs in HF care within a multidisciplinary team?
   1. What are their experiences with collaborative practice in the present?
   2. Which barriers do they experience in multidisciplinary collaboration?
   3. What could facilitate multidisciplinary collaboration?
   4. How do they envision the future? What are their needs towards multidisciplinary collaboration?
